# Persistent deficiency of mucosa-associated invariant T (MAIT) cells during alcohol-related liver disease

**DOI:** 10.1186/s13578-021-00664-8

**Published:** 2021-07-28

**Authors:** Yujue Zhang, Yuanyuan Fan, Wei He, Yi Han, Huarui Bao, Renjun Yang, Bingbing Wang, Derun Kong, Hua Wang

**Affiliations:** 1 Department of Gastroenterology, the First Affiliated Hospital of Anhui Medical University, Hefei, 230032 China; 2 Department of Oncology, the First Affiliated Hospital of Anhui Medical University, Hefei, 230032 Anhui China; 3 School of Basic Medical Sciences, Anhui Medical University, Hefei, 230032 Anhui China; 4 Department of Gastroenterology, Fuyang Hospital of Anhui Medical University, Fuyang, 236000 Anhui P.R. China; 5 Department of Emergency, the First Affiliated Hospital of Anhui Medical University, Hefei, 230032 China; 6 Inflammation and Immune Mediated Diseases Laboratory of Anhui Province, Anhui Medical University, Hefei, 230032 Anhui China

**Keywords:** Mucosal-associated invariant T cells, Alcohol-related liver disease, Severity, Inflammation, Apoptosis

## Abstract

**Background:**

Alcohol-related liver disease (ALD) is a major cause of chronic liver diseases. Inflammatory response is a basic pathological feature of ALD. Mucosal-associated invariant T(MAIT) cells are a novel population of innate immune cells, which may be depleted in various inflammatory diseases. However, the changes of MAIT cell in ALD remains unclear.

**Results:**

In this study, the levels of MAIT cell were significantly decreased in patients with alcoholic fatty liver disease, alcoholic cirrhosis, and mixed cirrhosis (alcoholic + viral). Furthermore, the reduction of circulating MAIT cells was correlated with liver function in patients with cirrhosis. Functional changes among circulating MAIT cells in patients with alcoholic cirrhosis, including increased production of IL-17A and perforin, and reduced production of TNF-α. Plasma cytokine and chemokine levels were quantified using multiple immunoassays and ELISA. Serum levels of chemokine IL-8 were correlated with MAIT cell frequency in patients with alcoholic cirrhosis. Moreover, no differences were observed in the expression of CCR6, CXCR6, and PD-1 in circulating MAIT cells of patients with alcoholic cirrhosis. The MAIT cells in patients with alcoholic cirrhosis were prone to apoptosis, which was promoted by IL-12, IL-18, and IL-8.

**Conclusions:**

Our findings indicate persistent MAIT cell loss during alcohol-related liver disease and suggest that MAIT cells can be promising indicator and therapeutic targets in ALD.

**Supplementary Information:**

The online version contains supplementary material available at 10.1186/s13578-021-00664-8.

## Introduction

Alcohol-related liver disease (ALD) is a major cause of chronic liver diseases worldwide. Its recent prevalence in China is similar to that in Europe and the United States [1]. Excess alcohol consumption can increase an individual’s susceptibility to viral infection and promote viral replication in vivo, thus promoting the occurrence of liver cirrhosis and liver cancer [2–4]. Moreover, binge drinking has gradually spread and become popular among young people [5]. The pathophysiology of ALD includes inflammation, gut dysbiosis, poor liver regeneration and immune dysfunction [6]. Immune cells, parenchymal cells, cytokines, and chemokines are involved in the inflammatory responses associated with ALD. Immune monitoring during alcohol consumption is of great significance for disease progression. Thus, it is imperative to better understand the pathogenesis of inflammatory responses in ALD to develop novel diagnostic and therapeutic targets.

Mucosal-associated invariant T (MAIT) cells are innate immune cells that are abundant in the liver [7]. MAIT cells can be activated by riboflavin metabolites derived from microorganism. Furthermore, MAIT cells also can be activated by IL-12, IL-18, IL-15 and IL-23. Therefore, MAIT cells can be considered both non-specific immune and acquired immune cells. MAIT cells reportedly have profibrogenic function [8], tissue repair [9] and antitumor [10] function. The diagnostic and prognostic value of MAIT cells in Type 1 diabetes [11] and cancer [12] have been reported. MAIT cells are also associated with the progression of HBV infection [13].

In ALD, although MAIT cells have anti-bacterial potency, they are depleted in both blood and liver [14]. It is of great importance to clarify the cause of MAIT cell loss for ALD therapy. Meanwhile, further exploration is required to identify specific biomarkers of MAIT cells in ALD. In the present study, we assessed whether MAIT cells could be used to identify patients with ALD at high risk for liver injury. We also explored the potential mechanism of MAIT cell loss associated with inflammatory cytokines and chemokines.

## Results

### Characteristics of patients

The clinical characteristics of patients are summarized in Table 1. Patients with alcoholic cirrhosis (53.6 ± 15.62 years of age) and those with mixed cirrhosis (52.21 ± 6.30 years of age) tended to be elderly individuals, while binge drinkers were mainly among the younger age group (28.34 ± 9.59 years). Moreover, male patients were more likely to drink than female patients. In some alcoholic cirrhosis patients, they still had excessive drinks. Liver biochemistry analyses, including alanine aminotransferase (ALT), aspartate transaminase (AST), total bilirubin (TBIL), γ-glutamyl transferase (GGT), alkaline phosphatase (ALP), and albumin (ALB), showed alterations in patients with cirrhosis. Interestingly, the proportion of neutrophils in binge drinkers was significantly increased.

**Table 1:** Clinical characteristics of patients

Parameter	Non drinker	ALD			Non-pathological alcohol drinker controls		
	Healthy control	Alcoholic fatty liver disease	Alcoholic cirrhosis	Mixed cirrhosis	Binge drinker	HDC	HBV liver cirrhosis
Number	116	4	48	33	34	11	7
Alcohol withdrawal (> 1 year)	–	0	23	33	–	–	–
Excessive drinks in the last 30d	–	–	10	0	34	11	–
Gender Male (%)	93(80.2%)	3(75%)	47(97.9%)	33(100%)	19(55.9%)	11(100%)	7(100%)
Age(year)	48.89 ± 11.21	40.25 ± 3.30*	53.6 ± 15.62	52.21 ± 6.30	28.34 ± 9.59^**^	51.36 ± 10.62	51.86 ± 3.18
ALT(U/L)	26.3 ± 30.54	71 ± 28.57	40.4 ± 52.42	32.25 ± 17.73	24.4 ± 5.77	51.73 ± 89.31	28.71 ± 16.04
AST(U/L)	21.08 ± 11.43	49 ± 15.28	59.29 ± 64.85^**^	39.88 ± 19.14^**^	23.6 ± 5.18	24 ± 10.68	24.43 ± 9.03
TBIL (μmol/L)	13.76 ± 5.89	21.63 ± 15.26	44.34 ± 49.55^**^	21.54 ± 19.63	8.94 ± 4.68	16.56 ± 14.56	19.24 ± 7.79
ALB(g/L)	41.75 ± 3.38	37.08 ± 9.40	31.63 ± 4.86^**^	32.76 ± 6.45^**^	46.82 ± 4.03	39.24 ± 4.98	39 ± 1.53
ALP(U/L)	78.10 ± 20.21	86.25 ± 13.57	140.16 ± 54.89^**^	124.63 ± 49.86^**^	66.75 ± 15.59	78.73 ± 17.64	83.14 ± 13.06
GGT(U/L)	31.71 ± 47.43	143.75 ± 108.69	149.78 ± 170.36^**^	70.16 ± 59.74^*^	23.75 ± 10.99	49.64 ± 58.96	26.14 ± 24.65
PT(s)	11.70 ± 1.02	13.1 ± 0.95	15.80 ± 3.21	15.39 ± 3.16	–	11.95 ± 0.7	12.13 ± 0.8
WBC (× 10^9^/L)	5.8 ± 1.59	7.87 ± 2.42	4.83 ± 2.22	4.0 ± 4.04	9.8 ± 3.64^**^	6.1 ± 0.73	4.99 ± 0.77
NEUT (%)	54.59 ± 7.57	71.45 ± 10.58	61.37 ± 15.98	58.73 ± 11.27	71.37 ± 12.45^**^	58.6 ± 8.64	53.03 ± 7.4
Neutrophil (× 10^9^/L)	3.22 ± 1.21	5.75 ± 2.56	3.05 ± 1.91	2.47 ± 2.98	7.22 ± 3.52^**^	3.63 ± 0.86	2.94 ± 1.2
Lymphocyte (× 10^9^/L)	2.37 ± 4.39	1.44 ± 0.51	1.18 ± 0.85	0.92 ± 0.81^*^	2.11 ± 0.99	1.87 ± 0.41	1.85 ± 0.39

All values provided as mean ± SEM; P-valued were determined by the one-way ANOVA(LSD), or Welch’s ANOVA test (Games-Howell test)

*ALT* alanine aminotransferase, *AST* aspartate transaminase, *TBIL* total bilirubin, *GGT* γ-glutamyl transferase, *ALP* alkaline phosphatase, *ALB* albumin, *PT* prothrombin time

All healthy controls (HCs) (n = 116) must exclude drinking history. ALD patients include alcoholic fatty liver disease (AFL) (n = 4), alcoholic cirrhosis (n = 48). Non-pathological alcohol drinker controls include binge drinkers (n = 34) and heavy drinkers without overt clinical evidence of liver disease (HDC) (n = 11). Patients with chronic HBV infection (n = 7) and mixed cirrhosis (n=33) were HBV DNA-negative.

^*^p < 0.05, **p < 0.01 (compared with blood from healthy individuals)

### *Circulating CD4* + *and CD8* + *MAIT cells are depleted in patients with alcoholic cirrhosis and mixed cirrhosis*

**Fig. 1:**
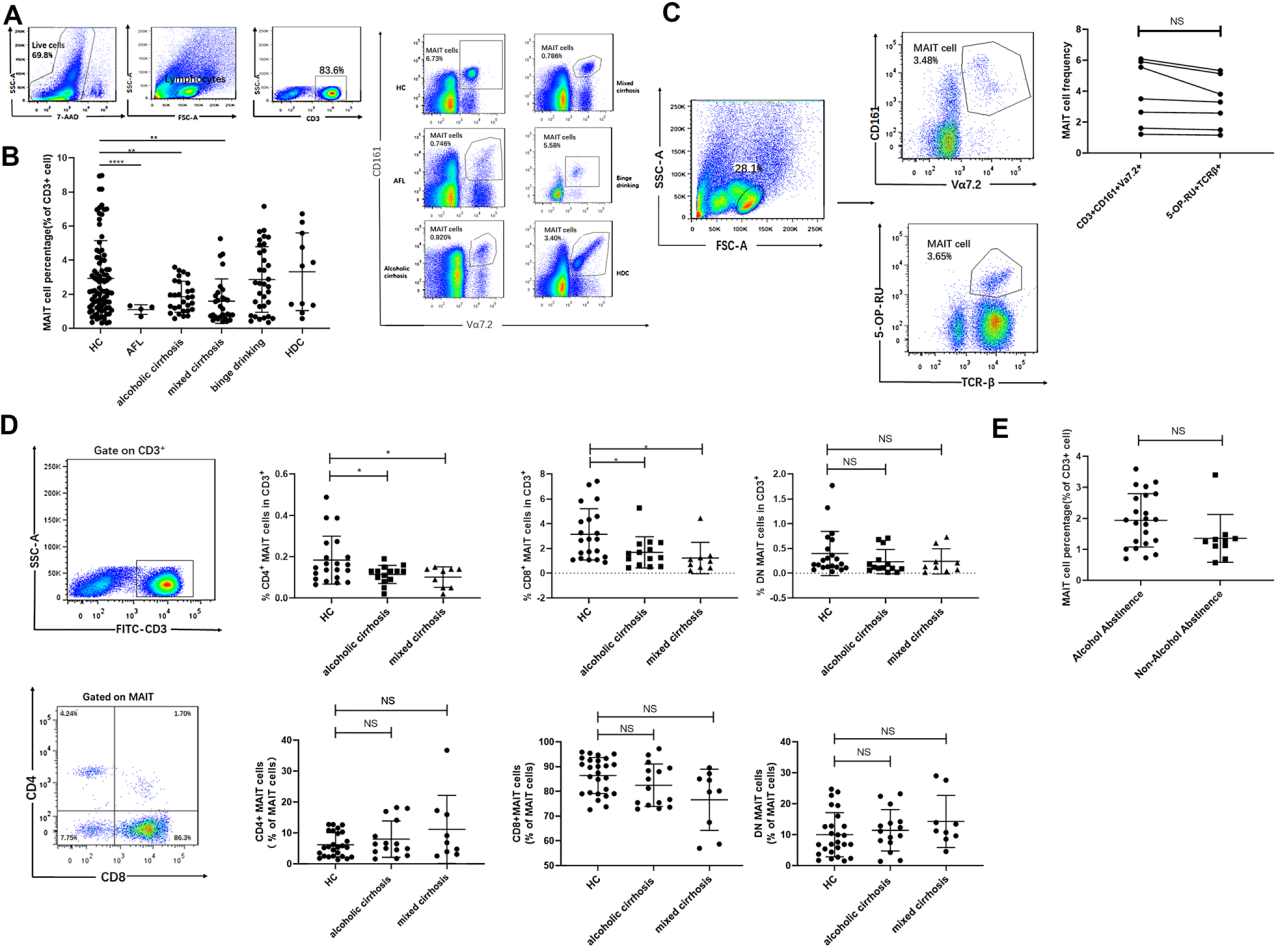
Circulating MAIT cell frequency was reduced in patients with ALD. **A** Gating strategy to identify mucosal-associated invariant T (MAIT) cells. Flow cytometry analysis of MAIT cells in human blood using CD3^+^CD161^+^Vα7.2^+^. **B** MAIT cells were depleted in patients with alcoholic fatty liver disease (n = 4), alcoholic cirrhosis (n = 29), mixed cirrhosis (n = 29), and in those who were binge drinker (n = 34) and heavy drinkers without overt liver diseases (n = 11), compared with HCs (n = 88). **C** MAIT cells were stained with the MR1 tetramer and T-cell receptor beta (TCR-β). No significant differences were observed between the two strategies (p = 0.065). **D** Percentage and constituent ratio of CD8^+^, CD4^+^, and double negative (DN) MAIT cells in patients with alcoholic cirrhosis, mixed cirrhosis, and HCs. **E** No significant differences were observed in MAIT cell frequency in alcoholic cirrhosis patients, who either did (n = 23) or did not (n = 10) abstain from alcohol consumption. Data are presented as the Mean ± SEM and analyzed by the paired-sample *t-*test, two-sample *t-*test, Welch’s analysis of variance (ANOVA) test, ANOVA test, LSD test, and Games-Howell test. HC, healthy control; HDC, heavy drinking controls (*p < 0.05; **p < 0.01; NS. p > 0.05)

Human MAIT cells were gated on CD3^+^CD161^+^Vα7.2^+^ lymphocytes (Fig. 1A). The frequency of MAIT cells was significantly depleted in patients with alcoholic fatty liver diseases (p < 0.001), alcoholic cirrhosis (p = 0.003) and mixed cirrhosis (p = 0.002), compared with HCs (Fig. 1B). However, no obvious reductions in the frequency of MAIT cells were observed among binge drinkers (p = 1) and heavy drinkers without overt liver diseases (p = 0.982). The frequency of MAIT cells had no difference in patients with alcoholic cirrhosis and mixed cirrhosis (p = 0.903). Furthermore, we found a significant reduction in the frequency of MAIT cells in patients with chronic HBV infection compared with healthy controls (p = 0.029) (Additional file 1: Fig. S1). In addition, no significant differences in the frequency of MAIT cells were observed between patients with chronic HBV infection and patients with mixed cirrhosis. We also used the 5-OP-RU tetramer and TCRβ to confirm our gating strategy and found no significant differences between the two gating strategies (p = 0.065) (Fig. 1C).

MAIT cells can be divided into subsets based on CD4 and CD8 expression [15]. In patients with cirrhosis, CD8^+^ and CD4^+^ MAIT cells among the T cells were both lower compared with HCs. Our further analysis indicated that the percentage of CD8^+^, CD4^+^, and CD4^−^CD8^−^(double negative, DN) cells among total MAIT cells were similar among patients with alcoholic cirrhosis, mixed cirrhosis, and HCs (Fig. 1D). In addition, no significant associations were noted between gender (p = 0.656) or age (p = 0.504) and MAIT cell frequency in HCs (Additional file 2: Fig. S2). We found there are no significant changes in the frequency of MAIT cells in alcoholic cirrhosis patients who had abstained from alcohol over 1 year, compared with patients who had still been excessive drinking (p = 0.076) (Fig. 1E). Thus, MAIT cells are depleted in chronic liver diseases, possibly related to long-term diseased state.

### Correlation between MAIT cell frequency and liver function in patients with cirrhosis

**Fig. 2:**
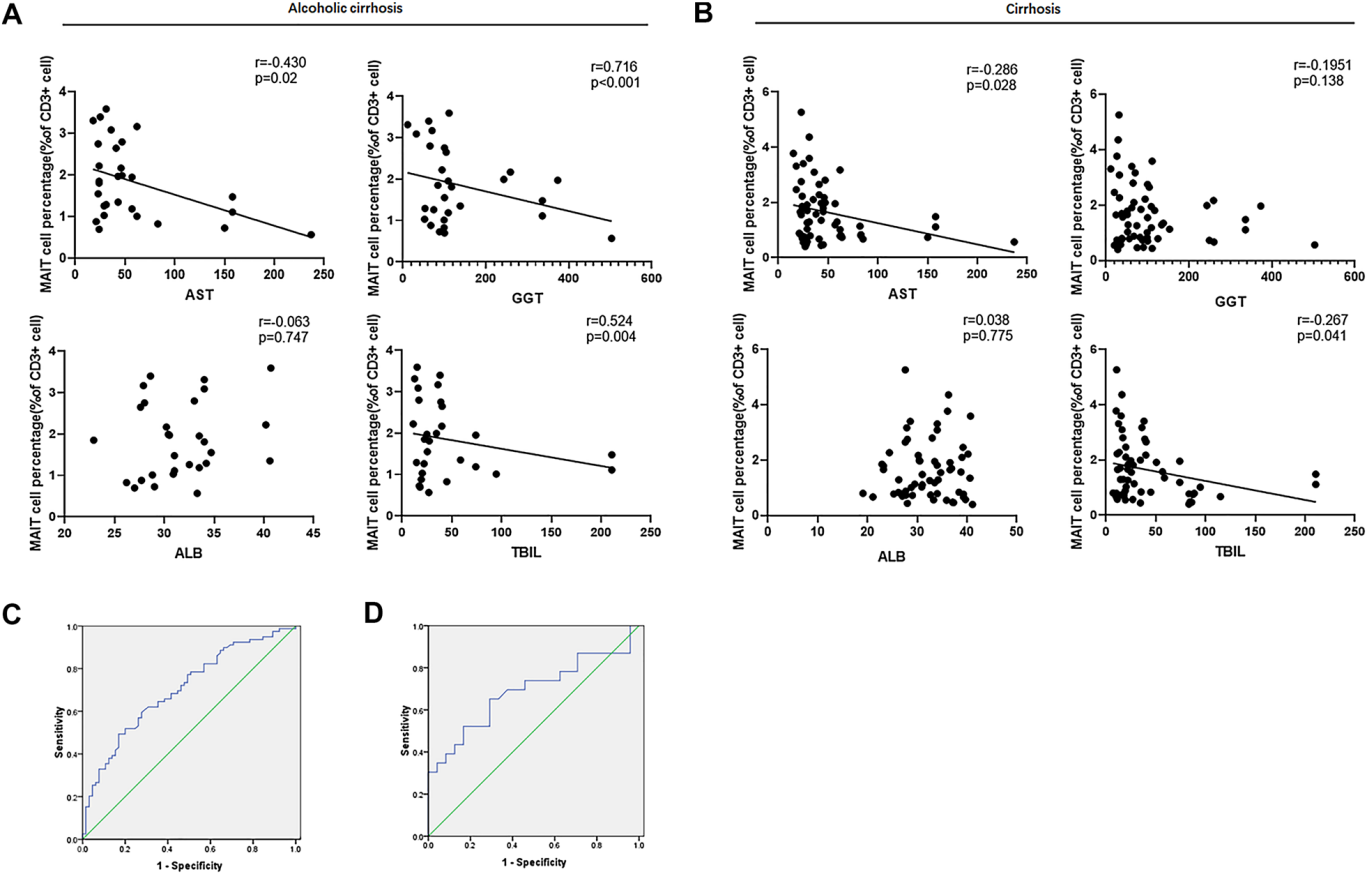
Circulating MAIT cell frequency correlated with liver function. **A** Spearman correlation between mucosal-associated invariant T (MAIT) cell percentages among CD3^+^T cells in patients with alcoholic cirrhosis, and levels of aspartate transaminase (AST), γ-glutamyl transferase (GGT), total bilirubin (TBIL), and albumin (ALB) (n = 29); **B** Spearman correlation between MAIT percentages among CD3^+^T cells in patients with alcoholic cirrhosis and those with mixed cirrhosis, and levels of AST, GGT, TBIL, and ALB (n = 60); **C** Receiver operating characteristic curve analysis of the diagnostic value of MAIT cell frequency in cirrhosis (AUC = 0.703, CI = 0.619–0.788, P < 0.001); **D** Receiver operating characteristic curve analysis of the diagnostic value of MAIT cell frequency for the severity of cirrhosis(Child–Pugh B/C)( AUC = 0.687,CI = 0.53–0.845,P = 0.028)

To assess the clinical significance of reduced MAIT cells in patients with alcoholic cirrhosis and mixed cirrhosis, we performed Spearman’s correlation analysis. Based on changes in clinical parameters presented in Table 1, we gained further insights regarding AST, GGT, TBIL, ALP, and albumin levels. Notably, a negative correlation was found between MAIT cell frequency and AST (p = 0.02), GGT (p < 0.001), and TBIL (p = 0.004) levels in patients with alcoholic cirrhosis, while no correlation was founded between ALB levels (p = 0.747) (Fig. 2A). In addition, a negative correlation was noted between MAIT cell frequency and AST, TBIL in all patients with cirrhosis. The correlation between MAIT cell frequency and GGT was not as evident as that in patients with alcoholic cirrhosis (Fig. 2B).

To assess the diagnostic ability of MAIT cells as it relates to validated markers of liver function, we constructed a receiver operating characteristic curve. We evaluated the diagnostic value of MAIT cells for cirrhosis, and determined the area under the curve, sensitivity, specificity, and cut-off values to be 0.703, 49.4%, 83.1%, and 2.805%, respectively (Fig. 2C). In order to discriminate between patients with cirrhosis and Child–Pugh A or Child–Pugh B or, C, we constructed a ROC characteristic, which yielded an area under the curve, sensitivity, specificity and cut-off values of 0.687, 65.2%, 70.8%, and 1.515%, respectively (Fig. 2D). Taken together, our data reveal a negative correlation between circulating MAIT cell frequency and liver function, and support the notion that MAIT can be a biomarker to evaluate liver injury in ALD.

### Activated MAIT cells display an altered cytokine profile and cytotoxic ability in patients with alcoholic cirrhosis

**Fig. 3:**
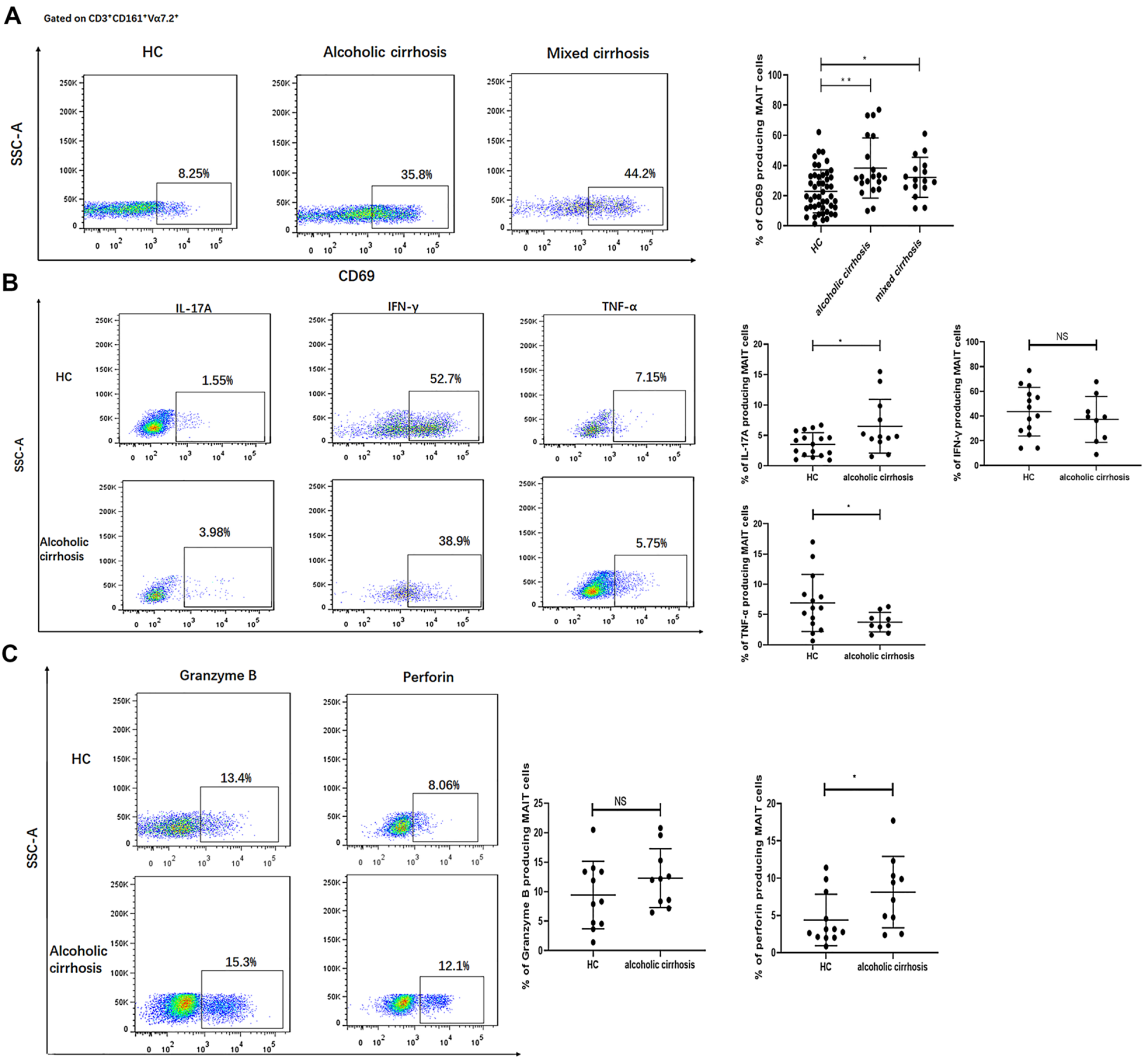
Phenotype and function of circulating MAIT cells in patients with alcoholic and mixed cirrhosis compared with healthy controls. **A** Representative dot plot of CD69. Comparison of CD69 on peripheral mucosal-associated invariant T (MAIT) cells in alcoholic liver disease (n = 20), mixed cirrhosis (n = 16), and healthy control (n = 47) (F = 7.418, p = 0.001). **B** Representative intracellular cytokine staining of MAIT cells from healthy control (HCs) and patients with alcoholic cirrhosis. Comparison of IL-17A, IFN-γ, TNF-α levels between HC (n = 14–17) and patients with alcoholic cirrhosis (n = 9–12). **C** Cytolytic capacity profile of MAIT cells in the blood from HCs and patients with alcoholic cirrhosis. Comparison of Granzyme B and perforin between HC (n = 11–12) and alcoholic cirrhosis (n = 10–11). Data were analyzed using the analysis of variance (ANOVA) test, LSD test, and two-sample *t*-test. (*p < 0.05; **p < 0.01; NS. p > 0.05)

We investigated the phenotype and effector functions of circulating MAIT cells in HCs and patients with ALD. We determined that circulating MAIT cells were activated in patients with cirrhosis by the elevation of CD69 levels (Fig. 3A). As T cell activation is associated with a change in effector function, we analyzed the ability of MAIT cells to produce cytokines and cytolytic proteins after PMA-ionomycin stimulation. Compared with HCs, IL-17 production was elevated in MAIT cells of patients with alcoholic cirrhosis (p = 0.02). The MAIT cells produced markedly lower levels of TNF-α in patients with alcoholic cirrhosis (p = 0.033). Moreover, MAIT cells showed higher perforin levels in patients with alcoholic cirrhosis (p = 0.046). In contrast, IFN-γ and granzyme B production were similar among MAIT cells in the blood samples of all subjects (Fig. 3B, 3C). Taken together, MAIT cells may be highly activated in patients with alcoholic cirrhosis, which results in an altered cytokine profile and cytotoxic ability.

### *Elevated IL-8 levels combined with elevated IL-12 and IL-18 levels promote apoptosis of MAIT cells *in vivo* and *in vitro

**Fig. 4:**
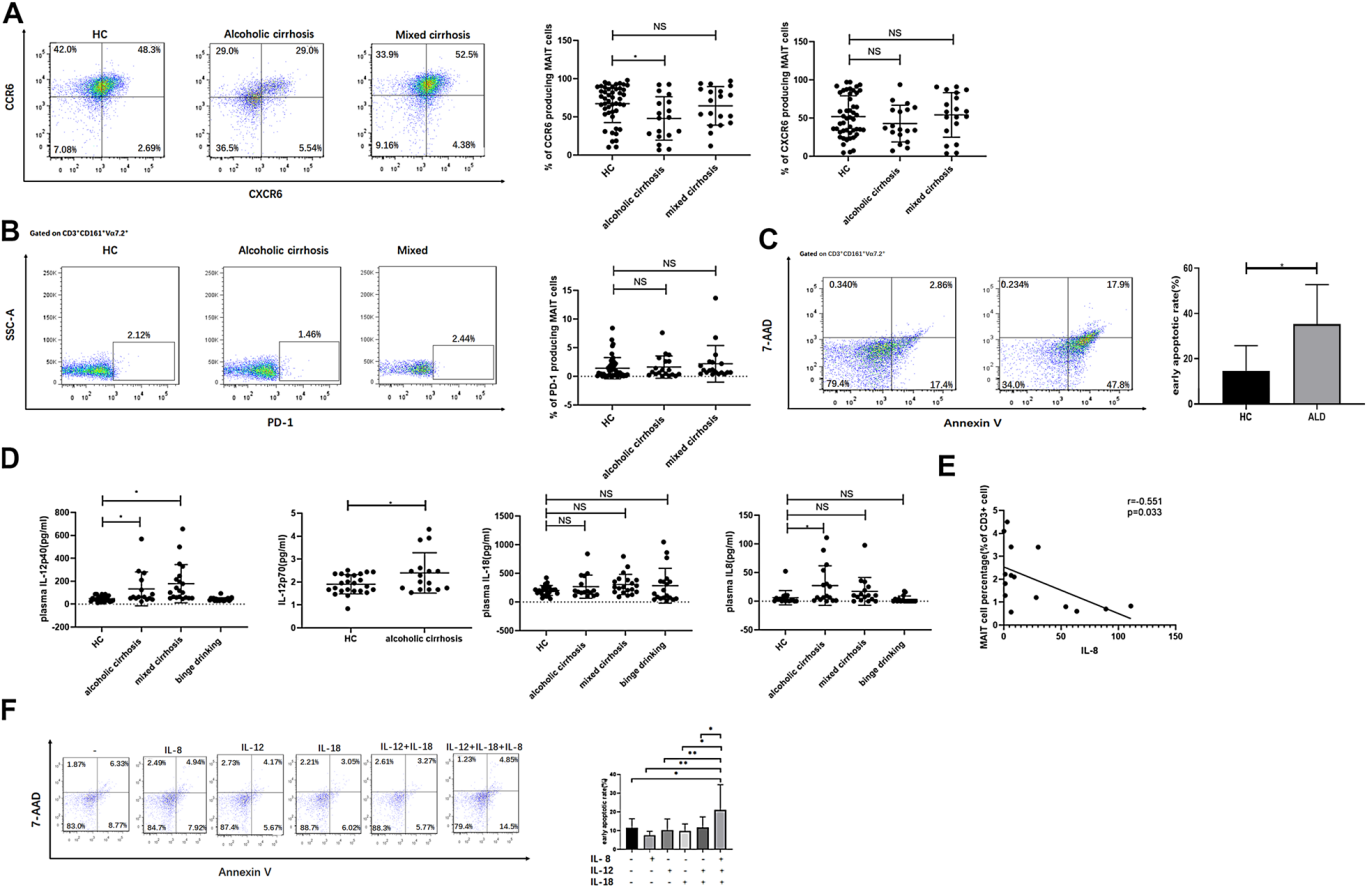
Inflammatory cytokines and chemokines promoted MAIT cell apoptosis. **A** Representative dot plot of CCR6 and CXCR6. Comparison of CCR6 and CXCR6 on peripheral mucosal-associated invariant T (MAIT) cells in alcoholic liver disease (n = 18), mixed cirrhosis (n = 19) and healthy control (n = 48). **B** Representative dot plot of PD-1. Comparison of PD-1 on peripheral MAIT cells in alcoholic liver disease (n = 19), mixed cirrhosis (n = 19) and healthy control (n = 48) (F = 0.816, p = 0.446). **C** Peripheral blood mononuclear cells (PBMCs) from patients with alcoholic cirrhosis (n = 5) and healthy controls (n = 6) were gated on MAIT cells and then stained with 7-aminoactinomycin D (7-AAD) and Annexin V. Percentage of early apoptotic (7-AAD^−^Annexin V^+^) cells were measured in healthy controls and patients with alcoholic cirrhosis. **D** Levels of IL-12p40, IL-12p70, and IL-8 were elevated in patients with alcoholic cirrhosis (n = 16) compared with healthy controls (n = 18–24). IL-18 was highly expressed in all groups. **E** Spearman correlation between MAIT percentage among CD3^+^T cells in patients with alcoholic cirrhosis with IL-8 levels (n = 15); **F** Representation of gating strategy showing 7-AAD and Annexin V after gating on MAIT cells in PBMCs from healthy humans, which were cultured with 50 ng/ml IL-12 or/ and IL-18 or/and IL-8 for 24 h. Early apoptosis was evidenced by the percentages of 7-AAD^−^Annexin V^+^ cells. Induced apoptosis in MAIT cells after exposure to IL-12, IL-18, and IL-8 stimulation for 24 h (n = 5). Data were analyzed by using the analysis of variance (ANOVA) test, two-sample *t*-test. (*p < 0.05; **p < 0.01; NS. p > 0.05)

We tried to determine the underlying cause for the reduced circulating MAIT cell frequency in patients with ALD. Previous studies have demonstrated that MAIT cells may be recruited to the liver in chronic liver diseases, which can be identified by elevated levels of CCR6 and CXCR6 [15]. However, we observed no increase in levels of CCR6 and CXCR6 on circulating MAIT cells in patients with alcoholic and mixed cirrhosis (Fig. 4A). Furthermore, previous studies have shown that apoptosis [17] or exhaustion [18, 19] may lead to the reduction in MAIT cell frequency. The expression of the immune checkpoint inhibitory molecule PD-1, often indicates T cell exhaustion [15, 19]. However, PD-1 expression on circulating MAIT cells showed no significant differences between patients with cirrhosis and HCs (Fig. 4B). As shown in Fig. 4C, MAIT cells were prone to apoptosis in patients with alcoholic cirrhosis (p = 0.034).

Cytokine-dependent pathways may contribute to MAIT loss [18, 20]. We further quantified cytokines and chemokines in HCs, patients with alcoholic cirrhosis and mixed cirrhosis, as well as binge drinkers (Table 2). As shown in Table 2, IL-12 and IL-23, which activate MAIT cells, were significantly increased in patients with cirrhosis. The concentration of IL-18, which is associated with the activation and exhaustion of MAIT cells [21, 22], was considerably elevated in all groups. In patients with alcoholic cirrhosis, the levels of IL-8 were significantly increased compared with those in HCs (Fig. 4D). Moreover, MAIT cells were inversely correlated with the levels of circulating IL-8 in patients with alcoholic cirrhosis (Fig. 4E). The PBMCs of healthy subjects were exposed to increasing doses of IL-8 and subjected to 24 h of culture. We found that different levels of IL-8 did not promote apoptosis in MAIT cells (Additional file 3: Fig. S3). Furthermore, we cultured PBMCs from HCs with IL-12, IL-18 and IL-8. Our results indicated that IL-12 and IL-18, combined with IL-8 can significantly enhance apoptosis of MAIT cells (Fig. 4F). Collectively, these data suggested that the inflammatory environment in alcoholic cirrhosis could promote the apoptosis of MAIT cells.

**Table 2:** Cytokine and chemokine concentrations

Parameter	HC (n = 18–24)	Alcoholic cirrhosis (n = 16)	Mixed cirrhosis (n = 16–20)	Binge drinking (n = 16–20)
TSLP (pg/ml)	5.77 ± 3.48	5.64 ± 4.89	2.11 ± 0.69^**^	1.79 ± 0.78^**^
IL-23 (pg/ml)	4.88 ± 2.49	9.47 ± 12.34^*^	9.62 ± 6.27^*^	4.27 ± 1.51
IL-12p40 (pg/ml)	50.21 ± 24.02	139.10 ± 151.39^*^	178.98 ± 166.71^*^	42.46 ± 14.87
IL-12p70 (pg/ml)	1.90 ± 0.42	2.15 ± 0.96^*^	–	–
IL-15 (pg/ml)	17.37 ± 23.80	59.09 ± 109.97	121.43 ± 139.51^*^	10.70 ± 42.48
IL-18 (pg/ml)	201.0 ± 85.4	267.37 ± 201.57	304.48 ± 177.26	283.61 ± 303.28
IL-8 (pg/ml)	3.22 ± 3.74	27.30 ± 34.52^*^	17.13 ± 24.24	3.28 ± 5.83
CXCL10 (pg/ml)	58.26 ± 29.22	126.24 ± 96.36^*^	77.40 ± 37.89	13.57 ± 9.64^**^
CXCL5 (pg/ml)	111.18 ± 168.80	15.74 ± 21.58^*^	16.45 ± 25.73^*^	82.79 ± 123.30
CXCL11 (pg/ml)	50.51 ± 27.56	59.16 ± 33.70^*^	31.58 ± 13.08	18.48 ± 6.87^**^
IL-7 (pg/ml)	222.01 ± 46.40	247.52 ± 43.39	119.58 ± 21.01^**^	175.22 ± 42.59^*^
IL-26 (pg/ml)	324.42 ± 122.10	240.47 ± 115.20	609.6 ± 177.29^**^	485.3 ± 160.26^**^
CCL11 (pg/ml)	50.51 ± 27.56	59.16 ± 33.70	31.58 ± 13.08	18.48 ± 6.87^*^
CCL2 (pg/ml)	294.88 ± 245.48	260.48 ± 169.80	152.33 ± 71.46^*^	99.41 ± 109.09^*^
CXCL9 (pg/ml)	26.89 ± 30	42.51 ± 55.05	29.70 ± 29.51	6.14 ± 9.83^*^

All values presented as the mean ± SEM. p-values were determined by the one-way analysis of variance (ANOVA) least significant difference (LSD), or Welch’s ANOVA test (Games–Howell test)

*HC* healthy controls, *TSLP* thymic stromal lymphopoietin, *IL*, interleukin, *CXCL* chemokine (C–X–C motif) ligand, *CCL* chemokine (C–C motif) ligand

*p < 0.05, **p < 0.01, (compared with blood samples from healthy controls)

## Discussion

MAIT cells protect against inflammation and infection in ALD by producing cytokines and cytotoxic effector molecules. Persistent and systemic inflammation may contribute to apoptosis of MAIT cells. To our knowledge, few researches focused on feasibility of MAIT cells as biomarkers for the severity of liver injury in ALD. Our findings show the potential role of MAIT cells in alcohol-related liver diseases and reveal a possible apoptosis-related mechanism of MAIT cell loss.

The activation of innate immunity and adaptive immunity contributes to liver inflammation and injury in ALD. MAIT cells are one of important lymphocyte subsets, which are considered to be an important bridge between innate and adaptive immunity. In the present study, we found no significant changes in the MAIT cells of binge drinkers or heavy drinkers without liver diseases. Rapid and excessive alcohol consumption had no effect on MAIT cells. Previous studies have confirmed that short-term alcohol abstinence does not restore the frequency or function of MAIT cells, while long-term alcohol abstinence only partially reverses abnormalities [14, 18]. Due to the limited samples, we have not identified whether alcohol consumption could affect MAIT cell function and this does require more in-depth investigations.

In the present study, alcohol abstinence had no effect on MAIT cells in patients with ALD. In addition, we found no evidence that the dual factors of a virus and alcohol lead to more severe conditions than alcohol alone on MAIT cell frequency. The changes in MAIT cells may have been related to persistent pathological changes associated with various diseased states. As a result, a significant reduction in MAIT cells was observed in patients with alcoholic fatty liver diseases, alcoholic cirrhosis and mixed cirrhosis. Furthermore, the frequency of MAIT cells was negatively correlated with AST and TBIL levels in patients with cirrhosis. Specifically, a negative correlation was noted between MAIT cells and GGT in patients with alcoholic cirrhosis. Although the sensitivity of MAIT cells as an initial screening tool is relatively low based on our ROC analysis, the circulating MAIT cell frequency can distinguish HCs from patients with cirrhosis. Moreover, MAIT cells could be used as indicators of the severity of liver disease. Further research will be required to establish the clinical utility of MAIT cells as markers for advanced liver diseases.

CD69 is an immune activation marker which promotes cell proliferation, cytokine production and cytotoxicity [23]. The expression of CD69 on MAIT cells was up regulated in ALD patients. Activated MAIT cells led to altered functions in patients with ALD. Many studies have confirmed that drug therapy could affect MAIT cell frequency and phenotype [8, 15]. Although the changes in circulating MAIT cells were not as informative as those in the liver, they did reflect the state of these cells in inflammatory diseases. IL-17 can regulate alcohol induced liver inflammation, fibrosis, bacterial dissemination and tumorigenesis [24, 25]. TNF-α is one of inflammatory cytokines for ALD development. TNF-α increased in both the circulation and liver of alcoholic hepatitis and have been shown to be associated with disease severity. Drugs for TNF-α depletion may have therapeutic properties in ALD [26]. The present study demonstrated that MAIT cells produce more IL-17A, and less TNF-α in patients with alcoholic cirrhosis. Furthermore, we assessed cytotoxic activity of MAIT cells which was associated with Granzyme B and perforin [27]. Increased perforin levels may indicate an increased antibacterial potency of MAIT cells in patients with alcoholic cirrhosis. In addition, the trends toward reducing levels of IFN-γ and increasing levels of Granzyme B may play a protective role in ALD. Altogether, we postulated that MAIT cells may promote anti-inflammatory and anti-bacterial responses in ALD.

Several factors may be responsible for MAIT cell depletion, including age [28], gender [15], redistribution [16], cell exhaustion and activation-induced cell death. However, we found no significant differences in gender and age in the MAIT cells of HCs. Neither did we observe any elevated expression of CCR6 or CXCR6 on circulating MAIT cells in patients with alcoholic cirrhosis or mixed cirrhosis. Owing to a lack of liver samples, we could not determine whether MAIT cells had migrated to the liver. Recent research has confirmed the migration of MAIT cells to ascitic fluid in patients with decompensated liver cirrhosis [29]. However, the most recent research has reported that MAIT cells could largely proliferate in situ in the infection organ but do not require CXCR6 for accumulation [30]. Further study of the tissue-resident phenotype of MAIT cells in cross-talk between different organs in patients with ALD may yield promising findings. Furthermore, MAIT cells in this cohort of patients with alcoholic cirrhosis and mixed cirrhosis showed no expression of the immune exhaustion marker, PD-1. In line with previous studies [8], we found that apoptosis may be one of reasons of MAIT cell loss in patients with alcoholic cirrhosis, possibly through activation-induced cell death.

In chronic HBV-infected patients, conjugated bilirubin can promote peripheral MAIT cell activation and apoptosis [13]. Bottcher et al. found long-term stimulation with IL-12 and IL-18 promote MAIT cell death in liver tissue [21]. Our data suggested MAIT cell depletion is a consequence of long-term systemic inflammation. MAIT cell activation-associated cytokines IL-12 and IL-23 were considerably increased in alcoholic cirrhosis. Interestingly, we observed high expression of IL-18 in all groups. IL-18 is a marker of hepatic steatosis and regulate the process of metabolic syndrome in NASH [31, 32]. Besides, IL-18 participate in antiviral therapy and pro-fibrogenic process in various chronic liver diseases [21, 33]. Li et al. [18] reported that IL-18 is negatively correlated with MAIT frequency in alcoholic hepatitis. We also observed that levels of IL-8 were elevated in patients with alcoholic cirrhosis and negatively correlated with MAIT cell frequency. However, we found that different doses of IL-8 had no effect on the apoptosis of MAIT cells. Considering the fact that MAIT cells become altered after activation, a lack of IL-8 receptors on activated MAIT cell is plausible. As previously described [14, 15, 29], we cultured PBMCs from HCs with IL-12, IL-18 and IL-8. We further confirmed that IL-12, IL-18 and a combination with IL-8 could induce apoptosis. The findings of the present study suggest that a cytokine-rich inflammatory environment promotes MAIT cell loss in patients with ALD. Nonetheless, persistent inflammation may be associated with a variety of cytokines and chemokines. It is also possible that additional cytokines besides those under investigation in the present study, are able to affect MAIT cells. We have only confirmed the effects of a few. Meanwhile, MAIT cell depletion may be related to other pathways of cell death. This process requires further investigation. In our study, we could not determine whether MAIT cells are depleted and occurred apoptosis in the liver. In the future research, we will make deep research in the liver of alcohol related liver disease.

## Conclusions

Reduced MAIT cells were negatively correlated with liver injury in patients with cirrhosis. MAIT cells could be used as a new indicator for liver injury in ALD. We demonstrated activated MAIT cells have anti-inflammatory and antibacterial effects. Increased levels of inflammatory cytokines and chemokines promoted the apoptosis of MAIT cells, which may be one of causes for MAIT cell loss. Our data highlighted the diagnostic and therapeutic potential of MAIT cells in ALD. Further studies are required to focus on the mechanisms of upregulation of MAIT cell frequency and the restoration of their function.

## Materials and methods

### Study subjects

This study was approved by the Ethics Committee of Clinical Research of Anhui Medical University. All participants gave informed consent. All blood samples were collected at The First Affiliated Hospital of Anhui Medical University. The study subjects included patients with alcoholic fatty liver disease (AFL) (n = 4), alcoholic cirrhosis (n = 48), mixed cirrhosis (n = 33), as well as those who were binge drinkers (n = 34), heavy drinkers without overt clinical evidence of liver disease (HDC) (n = 11), and those with chronic HBV infection (n = 7) and HCs (n = 116). The diagnosis of patients was based on previously described criteria [34–36]. Binge drinkers had rapid and excessive alcohol intake in 2 h and HDC patients had long-term drink history. AFL patients had short-term alcohol abstinence and mixed cirrhosis patients all had more than 1 year alcohol withdrawal. In addition to the criteria for alcoholic cirrhosis, patients with mixed cirrhosis also had HBV infection. All patients with HBV infection were HBV DNA-negative. Non-inclusion criteria included recent gastrointestinal bleeding, current bacterial infections, and immunomodulatory or antibiotic treatments. All participants with metabolic diseases, immune diseases, or cancer were excluded. All healthy controls must exclude drinking history. Clinical characteristics of the subjects are presented in Table 1**.**

### Blood samples

Peripheral blood samples were collected in EDTA tubes and separated into plasma and peripheral blood mononuclear cells (PBMCs). Plasma samples were stored at -80° C until further use. The PBMCs were immediately used for Flow cytometric analysis.

### PBMC isolation and culture

The PBMCs were isolated by density gradient centrifugation, using the Ficoll-Hypaque technique ( Solarbio, China), and freshly used for surface and intracellular staining to analyze frequency, phenotype, and function. To detect cytokine production and cytotoxicity markers, PBMCs were cultured in RPMI-1640 medium supplemented with 10% fetal bovine serum, and stimulated with phorbol 12-myristate-13-acetate(PMA)/ionomycin and Brefeldin A/Monensin (Multi sciences, China) for 4–6 h at 37 °C. Stimulated PBMCs were fixed and permeabilized using a FIX & PERM Kit (Multi sciences, China) to stain the intracellular markers. The PBMCs were further stimulated with IL-8, IL-12, or IL-18 (Novoprotein Scientific, China) at 50 ng/mL for 24 h.

### Flow cytometric analysis

Flow cytometry was performed as described using the following antibodies. The anti-CD3-FITC, anti-CD161-APC, and anti-TCR Vα7.2-Brilliant Violet 421(Biolegend, USA) were used to identify MAIT cells. Anti-CD4-PE/Cy7, anti-CD8-Perp/Cy5.5, anti-CCR6-PE/Cy7, anti-CXCR6-PE, anti-IL-17A-PE, anti-Granzyme B-PE/Cy7, and anti-Perforin-PE were obtained from Biolegend (USA). Anti-CD69-PE/Cy7, anti-PD-1-PE, anti-IFN-γ-PE/Cy7, anti-TNF-α-PE were obtained from BD (USA). Appropriate isotype control antibodies were used for each staining combination. We used 7-aminoactinomycin D(7-AAD) (BD Biosciences) staining to identify dead cells. Human MR1 tetramers loaded with a potent MAIT cell ligand-5-OP-RU or 6-FP were gifted from professor Li Bai. Apoptosis was allowed to progress and two channels were used to detect annexin V- FITC and 7-AAD (BD Biosciences) to determine the proportions of apoptotic cells. Data acquisition was achieved on BD FACS Verse system (BD Biosciences), and results were analyzed using FlowJo7.6 analysis software.

### Multiplex immunoassays

Human cytokines and chemokines were identified in the plasma of patients with alcoholic cirrhosis, mixed cirrhosis, as well as binge drinkers and HCs. The cytokines and chemokines (GM-CSF, IFN-α2, IL-1α, IL-1β, IL-11, IL-12p40, IL-12p70, IL-15, IL-18, IL-23, IL-27, IL-33, TSLP, CCL2/MCP-1, CCL3/MIP-1α, CCL4/MIP-1β, CCL5/RANTES, CCL11/Eotaxin, CCL17/TARC, CCL20/MIP-3α, CXCL1/GROα, CXCL5/ENA-78, CXCL8/IL-8, CXCL9/MIG, CXCL10/IP-10, CXCL11/I-TAC) were analyzed by flow cytometry bead-based immunoassay (LEGENDplex™ Human Cytokine Panel2/ Human Proinflammatory Chemokine Panel, BioLegend), according to the manufacturer’s instructions. Sample acquisition was achieved on a FACS Verse system equipped with the FACSuite software (BD Biosciences), and samples were analyzed with the LEGENDplex™ Data Analysis Software v8.0.

### Enzyme-linked immunosorbent assay (ELISA)

Circulating levels of IL-7 and IL-26 were not available in the above panel. Therefore, these cytokines in the plasma were quantified using enzyme-linked immunosorbent assay (ELISA; Jianglai Biotech, China).

### Statistical analysis

All data were presented as the mean ± SEM. The parametric Student’s *t*-test for paired or unpaired samples as appropriate, or the Mann–Whitney U test, one-way analysis of variance (ANOVA) least significant difference (LSD), Welch’s ANOVA test (Games–Howell test), or Spearman’s correlation were used to calculate statistical significance. The diagnostic performance of MAIT cells in identifying the severity of disease was assessed by analyzing receiver operating characteristic (ROC) curves. Statistical calculations were performed using the GraphPad Prism 8.0 and SPSS17.0 software. In all tests, p < 0.05 was considered statistically significant.

## Supplementary Information

**Additional file 1:** **Figure S1.** Mucosal-associated invariant T (MAIT) cells were depleted in patients with chronic HBV-infection (n = 7), alcoholic cirrhosis (n = 29), and mixed cirrhosis (n = 29) compared with healthy controls (HCs) (n = 88). Data are presented as the Mean± SEM and analyzed by the Welch’s ANOVA test and Games-Howell test. (*p < 0.05; **p < 0.01; NS. p > 0.05).

**Additional file 2:** **Figure S2.** Comparisons between peripheral mucosal-associated invariant T (MAIT) cell frequency with gender and age. Data were analyzed by the t-test and ANOVA test. (NS, p > 0.05).

**Additional file 3:** **Figure S3.** Representation of gating strategy showing 7-aminoactinomycin (7-AAD) and Annexin V after gating on mucosal-associated invariant T (MAIT) cells in peripheral mononuclear cells (PBMCs) from healthy humans, which were cultured in different concentrations of IL-8 for 24 h. Early apoptosis is represented by percentages of 7-AAD-Annexin V+ cells. Cell death is represented by percentages of 7-AAD+Annexin V+ cells. Data were analyzed using the ANOVA test and the least significant difference (LSD). (*p < 0.05; **p < 0.01).

## Data Availability

All data generated or analyzed during this study are included in this published article.

## Abbreviations

ALD: Alcohol-related liver disease
HC: Healthy control
HDC: Heavy drinker without clinical evidence of liver disease
MAIT: Mucosal-associated invariant T cells
PBMC: Peripheral blood mononuclear cells
ALT: Alanine aminotransferase
AST: Aspartate transaminase
TBIL: Total bilirubin
GGT: γ-Glutamyl transferase
ALP: Alkaline phosphatase
ALB: Albumin
