# Supplementary figures and images for: Persistent deficiency of mucosa-associated invariant T (MAIT) cells during alcohol-related liver disease
Source: Cell Biosci. 2021 Jul 28;11:148. doi: 10.1186/s13578-021-00664-8 (PMC8320031; doi:10.1186/s13578-021-00664-8)

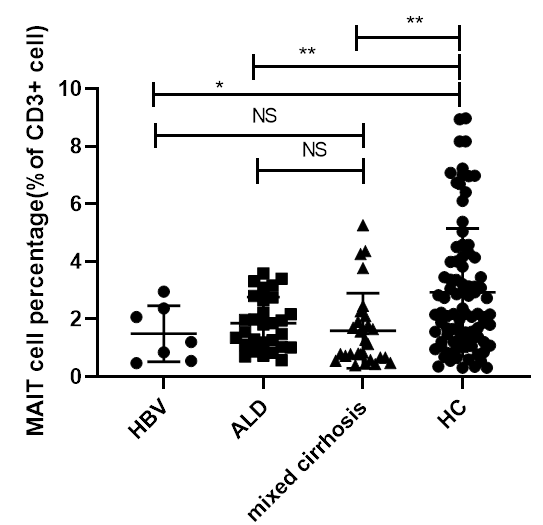

Supplement: Supplementary file 1 — Additional file 1: Figure S1. Mucosal-associated invariant T (MAIT) cells were depleted in patients with chronic HBV-infection (n = 7), alcoholic cirrhosis (n = 29), and mixed cirrhosis (n = 29) compared with healthy controls (HCs) (n = 88). Data are presented as the Mean± SEM and analyzed by the Welch’s ANOVA test and Games-Howell test. (*p < 0.05; **p < 0.01; NS. p > 0.05). [file 13578_2021_664_MOESM1_ESM.tif]

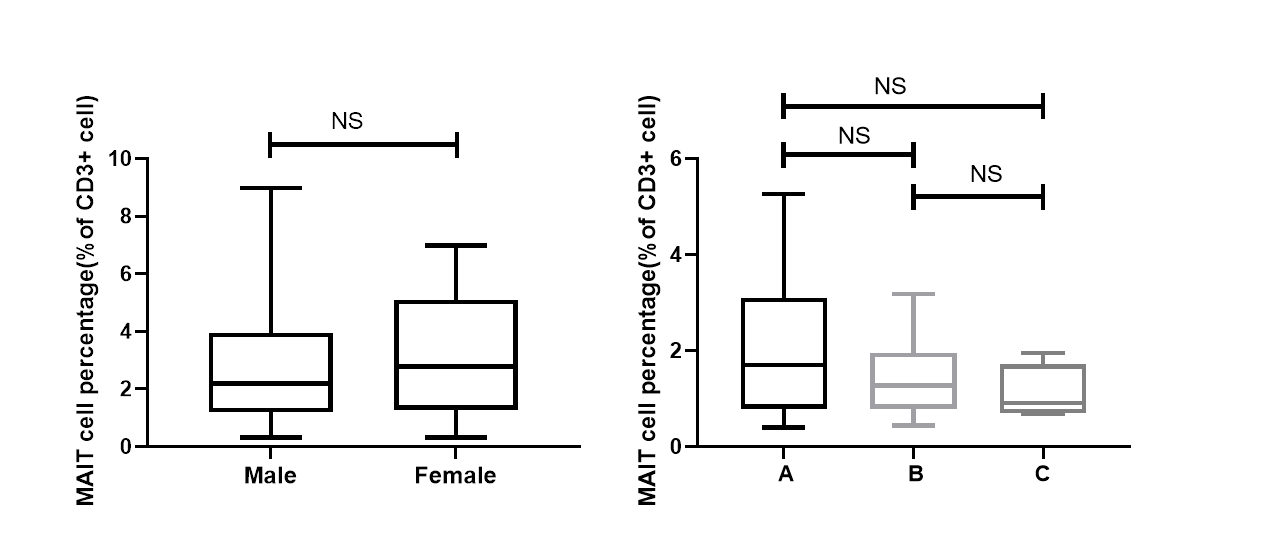

Supplement: Supplementary file 2 — Additional file 2: Figure S2. Comparisons between peripheral mucosal-associated invariant T (MAIT) cell frequency with gender and age. Data were analyzed by the t-test and ANOVA test. (NS, p > 0.05). [file 13578_2021_664_MOESM2_ESM.tif]

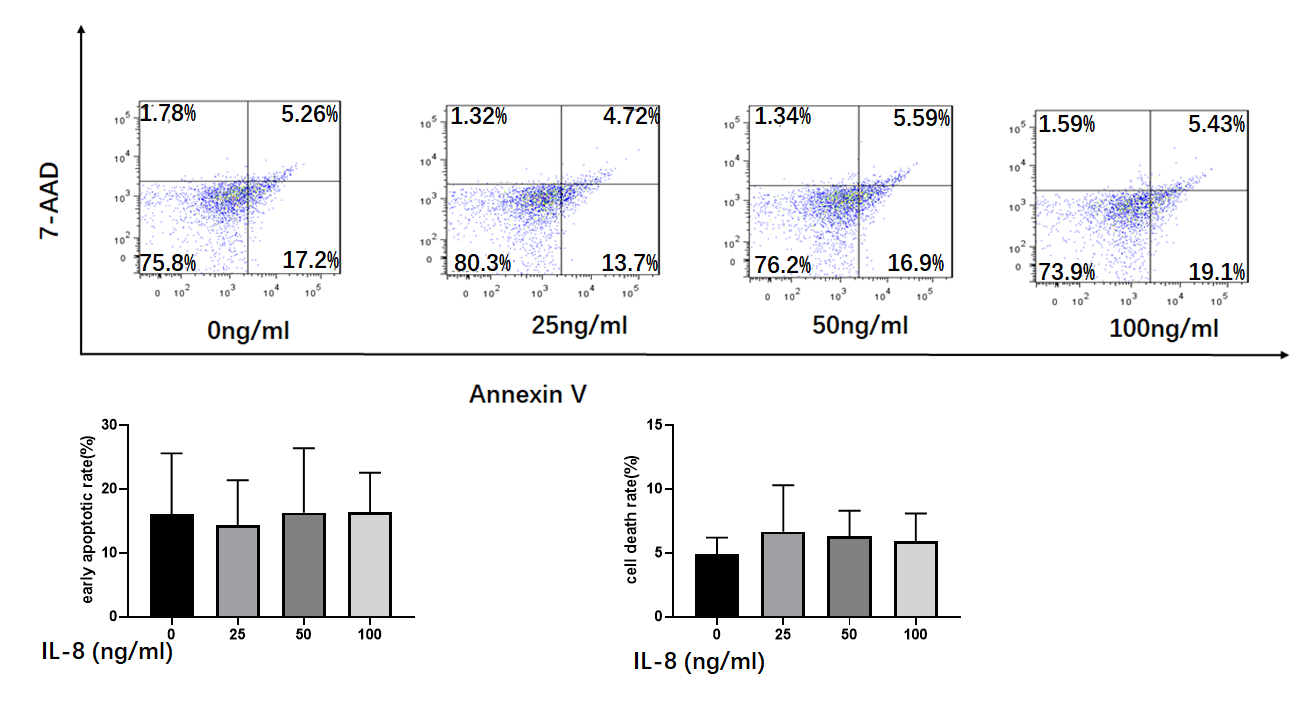

Supplement: Supplementary file 3 — Additional file 3: Figure S3. Representation of gating strategy showing 7-aminoactinomycin (7-AAD) and Annexin V after gating on mucosal-associated invariant T (MAIT) cells in peripheral mononuclear cells (PBMCs) from healthy humans, which were cultured in different concentrations of IL-8 for 24 h. Early apoptosis is represented by percentages of 7-AAD-Annexin V+ cells. Cell death is represented by percentages of 7-AAD+Annexin V+ cells. Data were analyzed using the ANOVA test and the least significant difference (LSD). (*p < 0.05; **p < 0.01). [file 13578_2021_664_MOESM3_ESM.tif]
